# Association Between Short Sleep and Body Mass Index, Hypertension Among Acute Coronary Syndrome Patients in Coronary Care Unit

**DOI:** 10.5539/gjhs.v7n3p134

**Published:** 2014-11-25

**Authors:** Elham Sepahvand, Rostam Jalai, Maryam Mirzaei, Marzieh Kargar Jahromi

**Affiliations:** 1 Department of paramedic, Lorestan University of medical science, Khoramabad, Iran; 2 Department of nursing, Kermanshah University of medical science, Kermanshah, Iran; 3 Department of nursing, Jahrom University of medical science, Jahrom, Iran; 4 Community Health Nursing, Jahrom University of Medical Science, Jahrom, Iran

**Keywords:** sleep disorders, body mass index, coronary disease, coronary care unit

## Abstract

**Introduction:**

Patients with coronary diseases admitted to special care unit often suffer from sleep disorders, which may cause physiological changes and adversely affect patient’s health. The relationship between sleep disorders and obesity is an important factor in studies on sleep disorders and other chronic diseases in all groups, including cardiovascular diseases. Understanding this relationship may increase the chance of progress in effective medical interventions in sleep disorders and obesity. This study was designed to evaluate the association between short sleep and Body Mass Index (BMI), hypertension among acute coronary syndrome patients.

**Materials and Methods:**

In this descriptive analytical study, 221 coronary patients admitted to coronary care unit and general wards were investigated. Data were collected through a researcher-made questionnaire whose validity and reliability had been confirmed. Data were analyzed with SPSS-16 software.

**Results:**

A total of 221 patients with acute coronary diseases (including myocardial infarction and angina pectoris) with a mean age of 61.27 years were studied, of whom 61.5% were male and 38.5% were female. A significant association was observed between short sleep and higher BMI (P=0.000). About half the patients (49.3%) had a history of hypertension, and sleep disorders were also significantly related to hypertension (P=0.006).

**Discussion:**

In this study, sleep disorders were patients’ main complaint. Researchers found that patients with less than 5 hours or more than 9 hours sleep at night were more likely to have hypertension compared to patients that slept 7-8 hours. Lack of sleep affects metabolism, and daily energy expenditure reduces with increased immobility. In this study, a significant relationship was observed between BMI and sleep duration among hospitalized patients in coronary care unit (P=0.000), and sleep disorders increased with higher BMI. Short of sleep increases sympathetic tonus, cortisol level, and activation of inflammatory pathways, impairing glucose metabolism and contributing to overweigh, increased visceral fat.

**Conclusion:**

Our findings suggest that poor sleep quality, is related to higher BMI and hypertension among acute coronary syndrome patients.

## 1. Introduction

As water and food, sleep is also an essential need of humans. Sleep is a reversible behavior mode with changes in level of consciousness and no response to environmental stimuli. In fact, sleep provides the time to modify, rebuild, and repair body systems. Also, repair, reorganization, memory build-up and learning occur during sleep (Neyse, Daneshmandi, Sadeghi Sharme, & Ebadi, 2011). A third of life is spent in sleep (Razmpa et al., 2009).

Many studies have shown that sleep is an essential need for improvement and survival of patients in intensive care units (Neyse, Daneshmandi, Sadeghi Sharme, & Ebadi, 2011; Hardin, 2009). Moreover, sleep plays a very important role in cardiovascular function, and sleep deprivation exacerbates anxiety, irritability, and increased myocardial oxygen demand (Neyse, Daneshmandi, Sadeghi Sharme, & Ebadi, 2011).Cardiac patients, especially those in Intensive care unit suffer disorders in sleep pattern, which may lead to physiological changes during sleep and adversely affect patient’s health (Tembo & Parker, 2009). Insomnia increases myocardial strength and speed of contraction and the need for supply of oxygen to heart. Sleep disorders cause release of epinephrine and norepinephrine and increased activity of sympathetic system and rising heart rate, respiratory rate and dysrhythmia, which intensify ischemia and infarction, ultimately leading to heart attack (Neyse, Daneshmandi, Sadeghi Sharme, & Ebadi, 2011).

There is a relationship between heart diseases, sleep, and heart disorders (Gay, 2010). Recent studies show that more than 28% of heart patients report poor sleep quality. Studies indicate that structure of sleep changes from increased light sleep (stage 1), reduced sleep with slow waves (stages 3 and 4) and REM. Their overall sleep duration varies from 2.1 to 8.8 hours, and it is not continuous. Additionally, 50% to 67% of their sleep is during night and 54% to 57% happen during daytime. This confirms that both their circadian rhythm and quality of their sleep have been affected (Beyer et al, 2010).

In recent decades, the prevalence of obesity and weight gain has been increasing in the world. Weight gain is associated with problems such as low self-esteem and heart diseases. Numerous studies have shown the relationship between reduced duration of sleep and increased BMI (Moraes et al., 2013). Not all studies show the relationship between obesity and sleep disorders, though (Vorona et al., 2005). Association of sleep disorders with obesity is an important factor in studies on sleep disorders and other chronic diseases in all groups, including cardiovascular diseases. Understanding this relationship may increase the chances of progress in effective medical interventions in sleep disorders and obesity (Hargens, Kaleth, Edwards, & Butner, 2013). Less than 6 hours sleep at night and frequent awakening at night are associated with increased likelihood of obesity (Vorona et al., 2005).

Many studies have shown that sleep disorders in patients with heart diseases is in the form of late sleeping, frequent waking at night, and early waking up before time (Spiegelhalder et al., 2011). Sleep-waking disorders are common in patients admitted to coronary care unit. Compulsory rest in bed that is necessary for acute heart patients may cause or intensify sleep disorders (Gottlieb et al., 2010; Grandner, Jackson, Pak, & Gehrman, 2011). Due to frequent awakenings, patients in coronary care unit have a discontinuous sleep pattern (Gottlieb et al., 2010). Many studies have investigated the relationship between sleep disorders and BMI in different groups of patients, but not in cardiac patients. Furthermore, although it has been confirmed in many studies that cardiac patients report sleep disorders, sleep disorders in these patients have not yet been investigated with a specific tool. Considering the relationship between BMI and sleep, and the effect of quality of sleep on quality of life in these patients, and since sleep disorder and quality of sleep in cardiac patients have not yet been investigated with a special sleep disorder questionnaire, thus, this study aims to investigate the relationship between sleep disorders in patients with acute coronary syndrome and their BMI.

## 2. Materials and Methods

This is a descriptive-analytical study conducted on patients with acute coronary syndrome admitted to coronary care unit in teaching hospitals in Kermanshah. A total of 221 cardiac patients that met study inclusion criteria were selected. Study inclusion criteria were diagnosis of coronary heart disease, aged 18 years and older, and willingness to take part. Patients with chronic diseases and unstable conditions (such as congestive heart disease), or with cognitive disorders (hearing or visual problems) or other problems that made participation difficult were excluded from the study. In the first stage, patients’ sleep pattern was assessed. Data were collected through a researcher-made questionnaire whose validity and reliability had been confirmed (Sepahvand, 2012). The questionnaire comprised two parts. Part one contained demographic questions about age, gender, education, occupation, history of admission in special care unit, history of previous diseases, and history of hypertension. The second part consisted of 25 questions about cardiac patients’ sleep disorder during hospitalization in ICU, with 5 options: “never”, “rarely”, “occasionally”, “often”, and “always” and scores from 0 to 4 respectively.

After selection, participants were allowed 3 days in coronary care unit to adjust to the environment and the effects on their sleep pattern and after necessary explanations and obtaining their consents, patients were provided with the questionnaire. Next, patients’ BMI was found by measuring their height and weight and was recorded in a special form for each patient. Data were analyzed with SPSS-16 software.

## 3. Results

A total of 221 patients with the mean age of 61.27 years were studied. No significant relationship was observed between age and BMI, 61.5% of patients were male and 38.5% were female. There was an insignificant relationship between gender and BMI (Table 1). Of participating patients, 48% were illiterate, 33.5% at high school level, 8.1% had high school diplomas, and 10.4% had higher education degrees. No relationship was found between education and BMI, either. 40.7% of patients had a history of diabetes, which had no significant relationship with BMI. 48.4% of patients had a history of hospitalization in special care units, which again had no relationship with BMI. There was a significant relationship between sleep disorders and BMI (P=0.000) (table 5). 49.3% of patients had a history of hypertension, which had a significant relationship with sleep disorders (P=0.006).

**Table 1:** Frequency distribution of demographic parameters, gender, history of diabetes and hypertension

Variable	Gender		History of hypertension		History of diabetes		History of admission in special care unit	

	Male	Female	Yes	No	Yes	No	Yes	No
Quantity	136	85	109	112	90	131	107	113
Percentage	61.5	38.5	49.3	50.7	40.7	59.3	48.4	51.1

**Table 2:** Mean score of quality of sleep in study subjects (221 patients)

Sleep quality indicators	Mean score
Initial insomnia	2.43±1.085
Frequent awakening at night	2.60±0.938
Terminal insomnia	2.50±0.934
Daytime fatigue	2.19±1.274
Duration of sleep	2.00±0.871
Quality of sleep	1.92±1.003
Total score	4.58±13.91

**Table 3:** Mean and standard deviation of parameters of: age, height, weight, and BMI in study subjects (221 patients)

Variable	Mean ± SD
**Age**	61.27±11.28
**Height**	1.68±0.102
**Weight**	74.49±12.31
**BMI**	26.54±4.17

**Table 4:** Status of BMI in study subjects (221 patients)

BMI	Quantity	Percentage
Less than 18.5	2	0.9
Between 18.5 and 24.9	80	37.9
Between 25 and 29.9	92	43.6
More than 30	37	17.5
Total	221	100

**Table 5:** Relationship between sleep disorders and BMI in study subjects (221 patients)

Sleep disorder	BMI									Total %

	>18.5		18.5<BMI<24.9		25<BMI<29.9		BMI>30			
	Quantity	%	Quantity	%	Quantity	%	Quantity	%	Quantity
No sleep disorder	1	5.3	12	63.2	5	26.3	1	5.3	19	100
Mild sleep disorder	0	0	44	39.6	55	49.5	12	10.8	111	100
Moderate sleep disorder	1	1.3	23	29.1	32	40.5	23	29.1	79	100
Severe sleep disorder	0	0	0	0	0	0	1	100	1	100
Total	2	1	79	37.6	92	43.8	37	17.6	210	100

Fisher’s exact test results showed a significant relationship between BMI and sleep disorders which increased with increasing BMI (P=0.000).

## 4. Discussion

Due to frequent awakenings, sleep has a discontinuous pattern in patients in coronary care unit. Studies indicate that structure of their sleep changes from increased light sleep (Stage 1), reduced sleep with slow waves (Stages 3 and 4) and REM. Their overall sleep duration varies from 2.1 to 8.8 hours, and it is not continuous. Recent studies show that more than 28% of heart patients report poor quality of sleep. In the present study, most patients reported initial insomnia and frequent awakenings at night (Table 2). A study by Johanssen et al. showed that initial insomnia happens in 21% of patients with myocardial infarction. In Johanssen study, initial insomnia and disrupted sleep were among common complaints in patients with myocardial infarction with sleep problems (Johansson, Karlson, Grankvist, & Brink, 2010). Gustafssen cites initial insomnia in patients with myocardial infarction, and reveals that falling asleep and continuity of sleep in cardiac patients is highly prevalent (Gustafsson U E, 1999). In a study by Tembo et al., all patients in special care unit suffered from frequent awakenings and severe reduction in REM (Hardin, 2009).

In the present study, duration of nocturnal sleep had reduced in most patients (161 patients, 72.9%). Otair et al. state that duration of nocturnal sleep in coronary care unit patients was less than normal (AL-Otair, AL-Shamiri, Bahobail, Sharif, & Bahamman, 2011). In a study by Hamazaki et al., duration of short sleep, less than 6 hours, was associated with the risk of cardiovascular diseases (Hamazaki et al., 2011). According to Drouot et al. study, patients in special care unit suffered sleep disorders in the form of reduction in overall duration of sleep and discontinuous sleep (Drouot, Cabello, Ortho, & Brochard, 2008). It seems that initial insomnia and frequent awakening reduce duration of sleep in cardiac patients.

In the present study, cardiac patients experienced terminal insomnia (134 patients, 60.6%). In the Gustafssen study, terminal insomnia was also observed in a large group of cardiac patients (Gustafsson, 1999). Mitamura used a questionnaire to study sleep disorders in patients in coronary care unit, with questions about disorders in onset and continuity of sleep. Terminal insomnia occurs due to reduced duration of REM following sleep disorders in cardiac patients (Mitamura, Nakamura, Yamamato, & Uchiyama, 2000).

In the present study, a significant relationship was observed between sleep disorders and hypertension (P=0.006). In a study by Morass et al., discontinuous sleep and reduced REM were associated with increased homocysteine and blood pressure (Moraes et al., 2013; Lusardi et al, 1999). Sleep disorders and deprivation cause activation of sympathetic system and rising blood pressure and heart rate through increased release of epinephrine and norepinephrine (Neyse, Daneshmandi, Sadeghi Sharme, & Ebadi, 2011).

Distribution of patients’ BMI is shown in Tables 3 and 4. In this study, a significant relationship was observed between BMI and sleep disorders in patients hospitalized in coronary care unit (P=0.000), and the increase in BMI led to an increase in sleep disorders. In Milia et al. study, a significant relationship was also found between reduced duration of sleep and obesity (Milia, Vandelanotte, &Duncan, 2013). Other studies also confirm that sleep disorders and reduced duration of sleep are related to high BMI (Moraes et al., 2013). Several mechanisms are involved in this such as reduced duration of sleep and increased waking leads to hunger and greater opportunity to eat, and thus increased calorie intake (Milia, Vandelanotte, & Duncan, 2013). Sleep shortage affects metabolism, and with increased immobility, reduces daily energy consumption. Additionally, Sleep shortage also can lead to increased sympathetic tonus, increased cortisol levels and activation of inflammatory pathways, impairing glucose metabolism and contributing to weight gain, increased visceral fat (Moraes et al., 2013; Milia, Vandelanotte, & Duncan, 2013). Spiegel et al. reported that reduced duration of sleep changes release of growth hormone and increases glucose level (Spiegel, Leproult, & Van Cauter, 1999). Meanwhile, sleep deprivation reduces release of apatite-regulating hormone, resulting in increased appetite (Chaput, Despre´s, Bouchard, & Tremblay, 2007; Mullington, Chan, & Van Dongen, 2003). All previous studies have investigated the relationship between sleep disorders and BMI in non-cardiac patients.

## 5. Conclusion

In conclusion, our findings suggest that poor sleep quality, is related to greater BMI and hypertension among acute coronary syndrome patients. Suggesting a mechanism to associate short sleep and increased body weight is not an easy task. Finally, more carefully designed longitudinal studies are necessary to confirm out findings.
